# Establishment of Efficient Genetic Transformation Systems and Application of CRISPR/Cas9 Genome Editing Technology in *Lilium pumilum* DC. Fisch. and *Lilium longiflorum* White Heaven

**DOI:** 10.3390/ijms20122920

**Published:** 2019-06-14

**Authors:** Rui Yan, Zhiping Wang, Yamin Ren, Hongyu Li, Na Liu, Hongmei Sun

**Affiliations:** 1 Key Laboratory of Protected Horticulture of Education Ministry and Liaoning Province, College of Horticulture, Shenyang Agricultural University, Shenyang 110866, China; yanrui2020@sina.cn (R.Y.); wangzp6119@163.com (Z.W.); RYM1258252544@163.com (Y.R.); lihongyu_syu@sina.com (H.L.); lnhhhhhh@163.com (N.L.); 2 National and Local Joint Engineering Research Center of Northern Horticultural Facilities Design and Application Technology, Shenyang 110866, China

**Keywords:** *Lilium*, genetic transformation, CRISPR/Cas9, somatic embryogenesis, *phytoene desaturase (PDS)*

## Abstract

*Lilium* spp. is a bulb flower with worldwide distribution and unique underground organs. The lack of an efficient genetic transformation system for *Lilium* has been an international obstacle. Because existing model plants lack bulbs, bulb-related gene function verification studies cannot be carried out in model plants. Here, two stable and efficient genetic transformation systems based on somatic embryogenesis and adventitious bud regeneration were established in two *Lilium* species. Transgenic plants and T-DNA insertion lines were confirmed by *β-glucuronidase (GUS)* assay, polymerase chain reaction (PCR) and Southern blot. After condition optimization, transformation efficiencies were increased to 29.17% and 4% in *Lilium pumilum* DC. Fisch. and the *Lilium longiflorum* ‘White Heaven’, respectively. To further verify the validity of these transformation systems and apply the CRISPR/Cas9 (Clustered Regularly Interspaced Short Palindromic Repeats (CRISPR)-associated protein 9) technology in *Lilium*, the *LpPDS* gene in the two *Lilium* species was knocked out. Completely albino, pale yellow and albino–green chimeric mutants were observed. Sequence analysis in the transgenic lines revealed various mutation patterns, including base insertion, deletion and substitution. These results verified the feasibility and high efficiency of both transformation systems and the successful application of the CRISPR/Cas9 system to gene editing in *Lilium* for the first time. Overall, this study lays an important foundation for gene function research and germplasm improvement in *Lilium* spp.

## 1. Introduction

*Lilium* is one of the most important horticultural crops, with high ornamental value and market demand [1,2]. For a long time, *Lilium* trait improvement and new variety creation have depended on long periods of hybridization and selection, while the application of more efficient genetic engineering and molecular breeding has not been possible. This barrier is mainly due to the lack of an efficient and mature genetic transformation system. The depth of basic theoretical research on *Lilium* has also been limited because the verification of most gene functions is conducted using *Arabidopsis*, *Nicotiana tabacum* and other model plants [3,4], which cannot reveal the relevant mechanisms in depth. With recent developments in molecular biology, especially the gene-editing technology represented by CRISPR/Cas9, rapid and targeted genetic improvement has been achieved in almost all grain crops and most horticultural crops, launching a new wave of molecular breeding [5]. Nevertheless, this powerful and universal technology requires a highly efficient and stable genetic transformation system [6,7]. Therefore, there is an urgent need to establish a more efficient, stable, and universal genetic transformation system in *Lilium*.

Compared with particle bombardment and other transgenic strategies in plants, the *Agrobacterium*-mediated method has many advantages, such as simple operation, low technical cost, high transformation efficiency and few transgene copies, making it the most common and universal transformation strategy. Since *Agrobacterium*-mediated genetic transformation of *Lilium* was first achieved in 1992 [8], several studies aiming to optimize this transformation system or to establish methods appropriate for different *Lilium* varieties, such as *Lilium longiflorum* [9], *Lilium* oriental [10,11], *Lilium formolongi* [12], and *Lilium tenuifolium* [13], have been carried out. However, due to the strong genotype dependence and low efficiency of stable transformation (up to 3%), poor genetic stability, and difficult regeneration of the receptor materials after transformation, this technique is still unable to meet the requirements of gene functional verification and trait improvement by genetic engineering.

Somatic embryos develop from a single cell [14], a process with the advantages of high genetic stability, low mutation and high regeneration rate [15,16,17]. Thus, they are considered good receptor materials for genetic transformation to obtain more stable transformed populations with a low rate of chimerism. Embryogenic calli can maintain their embryogenic state for a long time and are of great significance for genetic transformation [18]. Cohen found that the ability of loose embryogenic callus in suspension culture to accept exogenous genes was 50−70 times higher than that of common callus when applying gene-gun-mediated genetic transformation in *Lilium* [19]. In recent years, the use of embryogenic callus as a receptor material has become the focus of genetic engineering research. For instance, this technique has been successfully applied in *Nicotiana tabacum* [20], *Manihot esculenta* Crantz [21], *Curcuma longa* L. [22], *Helianthus tuberosus* L. [23], *Vitis vinifera* [24], *Zea mays* L. [25,26], *Oryza sativa* [27,28], and *Gossypium hirsutum* L. [29,30]. Thus, it may be practicable to establish an *Agrobacterium*-mediated genetic transformation system in *Lilium* based on somatic embryogenesis to effectively improve transformation efficiency and reduce the complexity of transgenic plant screening.

Because of its simple design principle and convenient operation, CRISPR/Cas9 has been rapidly adopted for basic theoretical research and crop improvement [31,32,33,34]. The first ornamental plant that was successfully edited by CRISPR/Cas9 is *Lotus japonicus* [35], a model leguminous plant, followed by *Chrysanthemum morifolium* [36]. To the best of our knowledge, the application of CRISPR/Cas9 genome editing technology in *Lilium* has not yet been achieved due to the limitations of genetic transformation. To quickly verify the effects of CRISPR/Cas9 in plants, the *PDS* gene encoding phytoene desaturase, a key enzyme of carotenoid synthesis, is often targeted as a reporter gene due to the obvious albinism and dwarfism caused by its loss of function as evidenced in *Arabidopsis* [37], *Citrus sinensis* [38], *Populus* [39], *Petunia hybrida* [40], *Nicotiana benthamiana* [41], *Medicago truncatula* [42], *Manihot esculenta* Crantz [43], *Triticum aestivum* L. [44] *Actinidia* Lindl. [33], *Brassica oleracea* [45], *Malus x domestica* Bork. and *Pyrus communis* L. [46].

This study focused on establishing two genetic transformation systems in two *Lilium* species through somatic embryogenesis and adventitious bud regeneration. These two genetic transformation systems, which each have distinct advantages, can greatly improve transformation efficiency and shorten the transformation period. The appropriate concentrations of antibiotics for screening positive transformants were determined, and key transformation conditions, including pre-culture, infection and co-culture duration, as well as the *Agrobacterium* culture concentration, were optimized. The T-DNA insertion lines were further confirmed by *β-glucuronidase (GUS)* assay, polymerase chain reaction (PCR) and Southern blot analysis. More importantly, we first attempted to apply CRISPR/Cas9 technology to *Lilium* by targeting the *PDS* gene, and the *pds* mutants of two *Lilium* species with albino phenotypes were verified by sequencing. These results proved the feasibility of these two transformation systems and the successful application of CRISPR/Cas9 to *Lilium*, which will contribute to basic theoretical research and molecular breeding in *Lilium*.

## 2. Results

### 2.1. Effects of Hygromycin (Hyg) and Cefotaxime (Cef)

In this assay, hygromycin (Hyg) sensitivity was measured by the browning and differentiation rates of the receptor materials on medium with different concentrations of Hyg. The results showed that 40 mg·L^−1^ Hyg could strongly inhibit the growth of cells around the embryogenic calli of *L. pumilum*, resulting in almost entirely browned and dead calli and a very low proliferation rate, while some adventitious buds were still produced on the scales of ‘White Heaven’ (Table 1). Thus, 30 mg·L^−1^ and 40 mg·L^−1^ Hyg were chosen to screen additional transformed embryogenic calli of *L. pumilum* and scales of ‘White Heaven’.

The results in Table 2 show that high cefotaxime (Cef) concentrations inhibited the proliferation and differentiation of the receptor materials. When the concentration of Cef was greater than 400 mg·L^−1^, the proliferation and differentiation rates of the receptor materials were too low, which affected normal growth. The results in Supplementary Figure S1 show that 400 mg·L^−1^ Cef could completely inhibit the growth of *Agrobacterium.* Therefore, the optimal concentration of Cef might be 400 mg·L^−1^, which could completely inhibit the growth of *Agrobacterium* without serious toxicity to the receptor materials.

### 2.2. Key Transformation Factors

**Figure 1:**
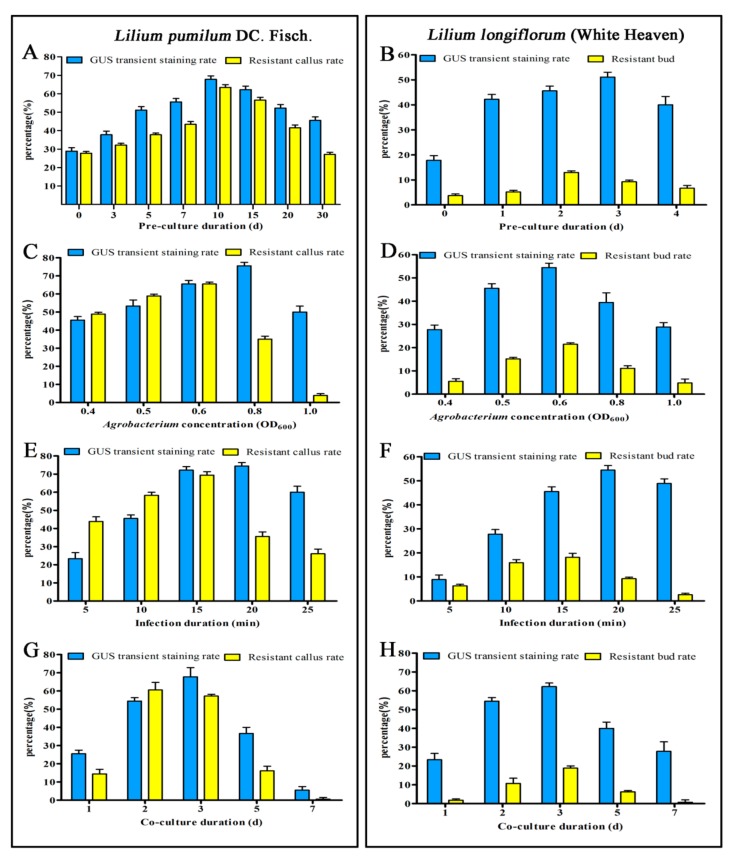
Parameters affecting transformation efficiency. **A** and **B** Effects of pre-culture duration on transformation efficiency. **C** and **D** Effects of Agrobacterium concentration on transformation efficiency. **E** and **F** Effects of infection duration on transformation efficiency. **G** and **H** Effects of co-culture duration on transformation efficiency. Left results for *Lilium pumilum* DC. Fisch.; right results for ‘White Heaven’. All data are expressed as means ± standard deviation of triplicate samples. d: days.

In this experiment, the two transformed *L. pumilum* and ‘White Heaven were pre-cultured for different durations, and the effects of different pre-culture durations on *Lilium* transformation under the same conditions were investigated. As shown in Figure 1A,B, the expression rate of *GUS* and the proliferation and differentiation rates of recipient materials in the calli and scales infected without pre-culture were very low. The *GUS* expression rate and resistant callus rate at 10 days of embryogenic callus pre-culture were 66.67% and 63.33%, respectively. The *GUS* expression rate at 3 days of scale pre-culture was the highest, with 50%. After 4 days of pre-culture, the resistant bud rate began to decrease. Therefore, we concluded that pre-culture of the embryogenic calli of *L. pumilum* for 10 days might be more conducive to the proliferation and differentiation of transformed receptor materials and 3 days might be the most suitable for ‘White Heaven’ scales.

Bacterial concentration is related to the growth status of *Agrobacterium* and the resistance of the cells, which both affect transformation efficiency. As shown in Figure 1C,D, when OD_600_ was 0.8, embryogenic calli attained the maximum *GUS* expression rate, but its proliferation was seriously affected. When the OD_600_ was 0.6, the expression rate of *GUS* and the resistant bud rate for ‘White Heaven’ scales were the highest. Therefore, combining the expression rate of *GUS* with the proliferation and differentiation ability of the receptor materials, OD_600_ = 0.6 was chosen as the optimal concentration for transformation.

Infection duration is an important factor affecting the efficiency of genetic transformation. The results showed that the rate of *GUS* expression in embryogenic callus and scales reached the highest level after 20 min of infection, but the proliferation of resistant callus and the regeneration of resistant buds were seriously affected (Figure 1E,F). Thus, an overly long infection duration might mean that *Agrobacterium* could not be easily cleared, thereby resulting in over-proliferation and mass necrosis of the receptor materials. However, 15 min of infection led to a higher *GUS* expression rate and stronger differentiation ability of the receptor materials. An excessive infection duration might result in too high a toxicity of *Agrobacterium* to the receptor materials, while too short a duration might mean an incomplete infection process. Therefore, 15 min might be the ideal duration for *Agrobacterium* infection.

Co-culture is an important period in which T-DNA can be transferred into plant cells. Thus, co-culture duration also has a large effect on transformation efficiency. The expression rate of *GUS* was the highest after 3 days of co-culture, while *Agrobacterium* overgrowth after 5 days of co-culture led to receptor material browning and death (Figure 1G,H). Therefore, the optimal co-culture duration might be 3 days.

### 2.3. Regeneration of Transgenic Plants

**Figure 2:**
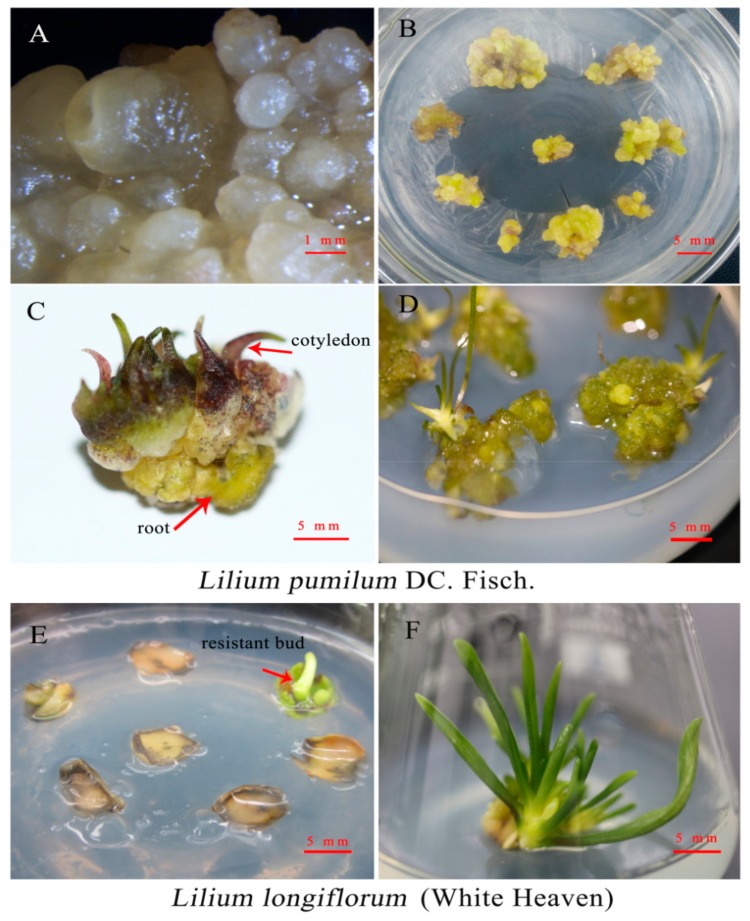
Plant tissues at different stages of *Agrobacterium*-mediated transformation of *Lilium*. **A**–**D** Formation of transformed plants of *Lilium pumilum* DC. Fisch. **A** New embryogenic cells of resistant callus. **B** Resistant callus germination. **C** Resistant buds formed during germination. **D** Transformed plants. **E**–**F** Formation of transformed plants of ‘White Heaven’. **E** Resistant bud. **F** Transformed plants of ‘White Heaven’.

In this study, the transformation of *L. pumilum* was accomplished through the somatic embryo pathway. After 4 weeks of screening and culture, resistant embryos were produced in resistant callus (Figure 2A), and untransformed embryos gradually browned and died. The necrotic tissues at the base of the calli were removed, and the somatic embryos were transferred to germination medium. After 2 weeks, the resistant calli turned green (Figure 2B) and continued to grow for 2 weeks to form mature somatic embryos with cotyledons and roots (Figure 2C). Then, the mature somatic embryos were transferred to culture on MS (Murashige-Skoog) medium and developed into intact plants after 2 weeks (Figure 2D). The transformation of ‘White Heaven’ was accomplished through adventitious bud regeneration. After 2 weeks of resistance screening, the adventitious buds (Figure 2E) could be transferred to MS medium for 15 days to form complete plants (Figure 2F).

### 2.4. β-Glucuronidase (GUS) Histochemical Assay

**Figure 3:**
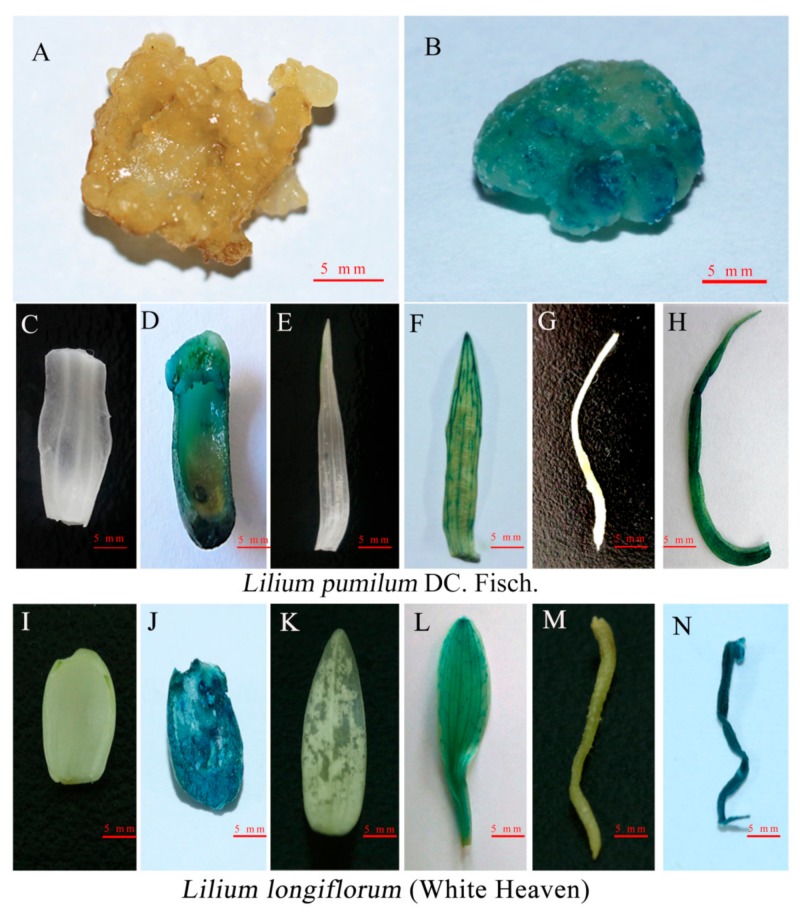
*β-glucuronidase (GUS)* histochemical assays of transgenic plants. **A**–**H** *GUS* histological staining results of *Lilium pumilum* DC. Fisch. **A** Untransformed embryogenic callus. **B** Transformed embryogenic callus. **C**–**H** show the scale, leaf, and root, respectively (**C**, **E** and **G** are untransformed plant; **D**, **F** and **H** are transformed plant). **I**–**N** *GUS* histological staining results for the scale, leaf, and root of ‘White Heaven’ (**I**, **K** and **M** are untransformed plant; **J**, **L** and **N** are transformed plant) (bars = 5 mm).

Because the *GUS* gene contains an intron sequence in pCAMBIA1301, it can be effectively expressed in plant cells but not in *Agrobacterium*, so the expression of *GUS* is a reliable indicator of plant transformation. *GUS* histochemical staining was used to identify transformed calli, scales, leaves, and roots. Blue staining was observed in different tissue parts (Figure 3), confirming the stable expression of *GUS* in the whole plant, while no blue staining was observed in the untransformed plants.

### 2.5. Polymerase Chain Reaction (PCR) and Southern Blot Analysis

**Figure 4:**
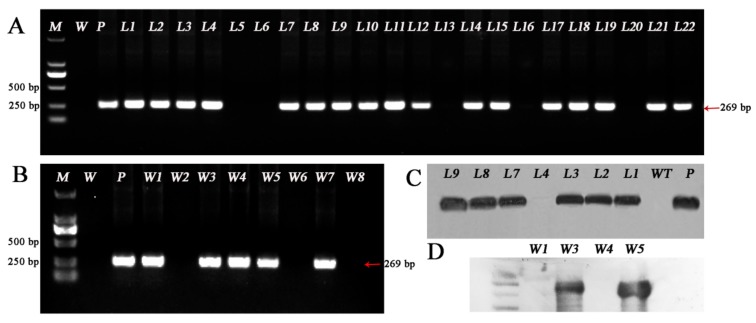
Polymerase chain reaction (PCR) analysis of transgenic plantlets using the *GUS* gene. **A** Only 22 *Lilium pumilum* DC. Fisch. lines are shown **B** Only 8 White Heaven’ lines are shown, and others no band was amplified. Lane M, DL2000 DNA marker; lane W, wild-type plant; lane P, plasmid control; lanes L1–22 are GUS-positive lines of *Lilium pumilum* DC. Fisch. and lanes W1–8, are GUS-positive lines of ‘White Heaven’. **C**, **D** Southern blot analysis of transformation. lane WT, wild-type plant; lane P, plasmid control; lanes L1-9, Only 9 PCR-positive transgenic lines of *Lilium pumilum* DC. Fisch. are shown. lanes W1, W3, W4 and W5, PCR-positive transgenic lines of ‘White Heaven’. W7 no band was amplified.

The genomic DNA of *GUS*-stained positive plants was extracted, and genomic DNA samples from the untransformed plants and plasmid pCAMBIA1301 were used as negative and positive controls, respectively. PCR amplification was performed using the specific primer GUS-F/R and detected by agarose gel electrophoresis. The PCR product of the *GUS* gene (269 bp) was amplified from 35 lines out of 120 for *L. pumilum* and 5 lines out of 125 for ‘White Heaven’, while no band was amplified from nontransformed plants, thus the transformation efficiencies were 29.17% (35/120) and 4% (5/125) in *L. pumilum* and ‘White Heaven’, respectively (Figure 4A,B).

To further verify the integration of the target gene into the *Lilium* genome, Southern blot analysis of PCR-positive transgenic lines was performed. The results showed that hybridization signals were observed in 15 lines out of the 35 lines (42.86%) of *L. pumilum*, 2 lines out of the 5 lines (40%) of ‘White Heaven’ and the plasmid control plants, while hybridization signals were not observed in the nontransgenic control plants (Figure 4C,D), thus confirming that the *GUS* gene had been integrated into the *Lilium* genome. The stable transformation efficiency reach 12.5% (15/120) and 1.6% (2/125) in *L. pumilum* and ‘White Heaven’, respectively.

### 2.6. Phenotype of Transformed Plants with pBUE-LpPDS

**Figure 5:**
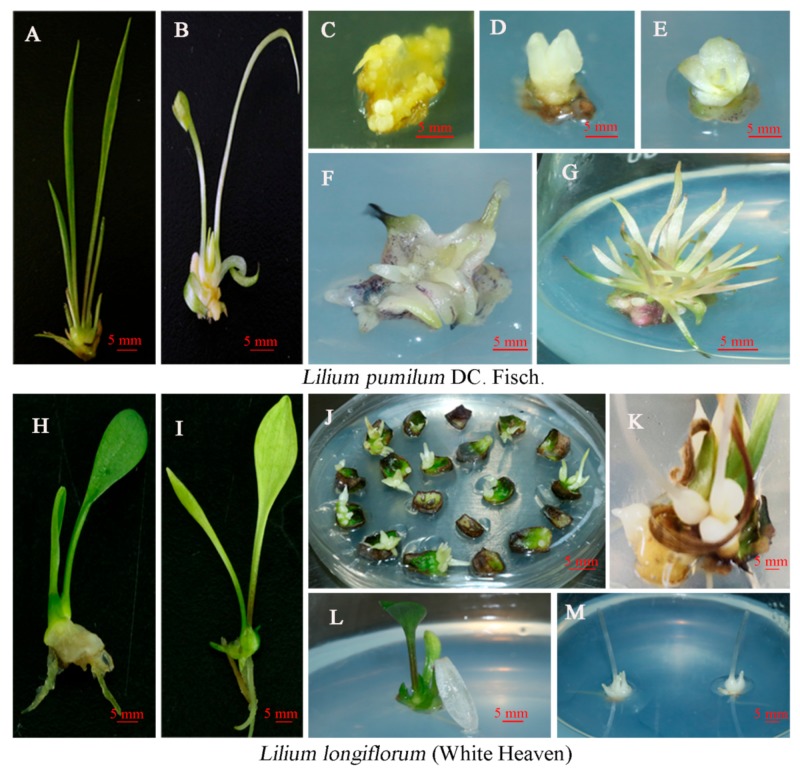
Phenotypes of *LpPDS* mutants after transformation with the CRISPR/Cas9 system. A–G show *Lilium pumilum* DC. Fisch. **A** Nontransgenic plant. **B** Yellowing mutant. **C** Resistant somatic embryos. **D** Mature embryo. **E**–**F** Dwarf mutants. **G** Completely albino plants. **H**–**L** show ‘White Heaven’. **H** Nontransgenic plant. **I** Yellowing mutant. **J** Resistant buds. **K** and **L** Mixed green and white leaves. **M** Completely albino plants (bars = 5 mm).

The inactivation of *PDS* gene function will lead to the destruction of chlorophyll under light conditions, which will turn green tissues white. In this study, plants with obvious phenotypic changes were observed 60 days after transformation of *L. pumilum* and at 15 days in ‘White Heaven’. A total of 30% (45/150) of *L. pumilum* and 5.17% (31/600) of ‘White Heaven’ with resistance and obvious phenotypes were obtained (Figure 5). These lines were divided by phenotype into groups with completely white leaves (Figure 5G,M), yellow leaves (Figure 5B,I), and white-and-green leaves (Figure 5K,L) leaves. The yellow phenotype rate of *L. pumilum* was 24.67% (37/150), and the completely white phenotype rate was 5.33% (8/150). The yellow phenotype rate of ‘White Heaven’ was 4% (24/600), the white-and-green phenotype rate was 0.8% (5/600), and the completely white phenotype rate was 0.3% (2/600). We conducted in-depth studies on the plants with significant phenotypes, and did not further explore those with no obvious phenotype.

### 2.7. Molecular Analysis of CRISPR/Cas9-Induced Mutations in LpPDS

**Figure 6:**
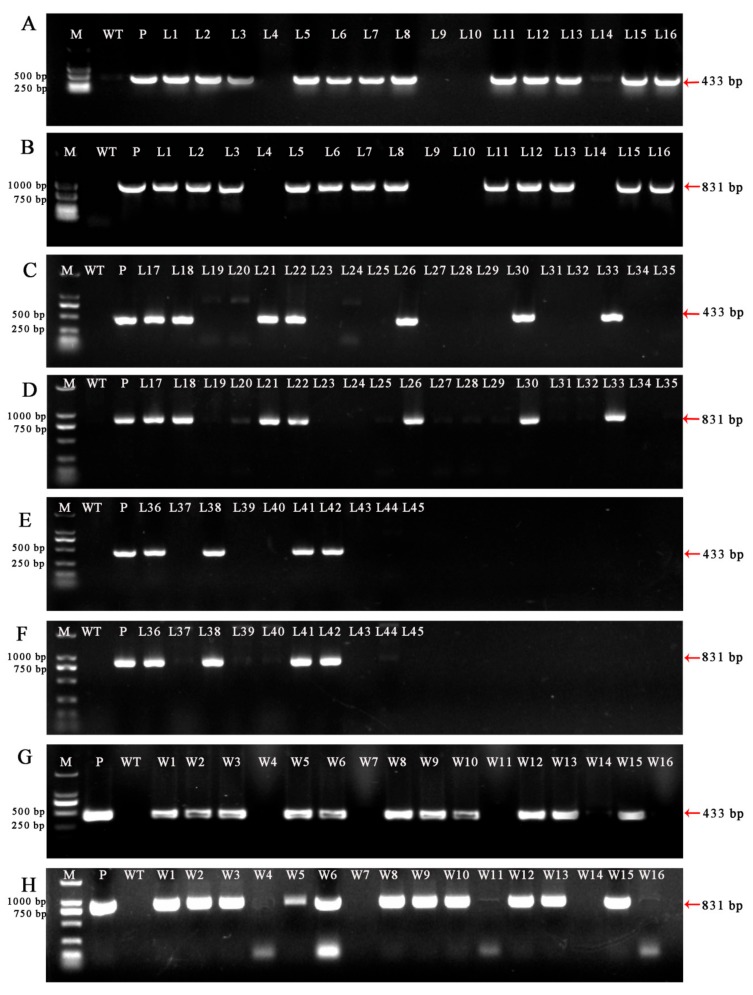
PCR analysis of plants with obvious albino phenotypes using the *Bar* gene and vector-specific primers. **A**, **C** and **E** PCR results for the *Bar* gene (433 bp) of *Lilium pumilum* DC. Fisch. **G** *Bar* gene (433 bp) of ‘White Heaven’. **B**, **D** and **F** PCR fragment of a partial sgRNA expression cassette (831 bp) of *Lilium pumilum* DC. Fisch. **H** PCR fragment of partial sgRNA expression cassette (831 bp) of ‘White Heaven’. Lane M, DL2000 DNA marker. lane WT, wild-type plant. lane P*,* plasmid control. **A**–**F**: Lanes 1–45, obvious albino phenotypes plants of *Lilium pumilum* DC. Fisch. **G** and **H**: Lanes W1–16, obvious albino phenotypes plants of ‘White Heaven’, Only 16 lines are shown, and others no band was amplified.

Genomic DNA was extracted from the regenerated plants and amplified by PCR using the selection marker gene *Bar* (Basta resistance gene) and sgRNA expression cassette primers (OsU3-FD3 and TaU3-RD). Target fragments of 433 bp (Figure 6A,B) and 831 bp (Figure 6C,D) were amplified using a vector plasmid (Figure 6 lane P) with the target gene as a positive control. DNA from wild-type plants (Figure 6 lane WT) did not result in amplification of the target fragments, while resistant plants showed a fragment of the same size as the positive control plants. Among the resistant plants with obvious phenotype, the PCR positive rate of *L. pumilum* was 51.11% (23/45), and the positive rate of ‘White Heaven’ PCR was 35.45% (11/31).

**Figure 7:**
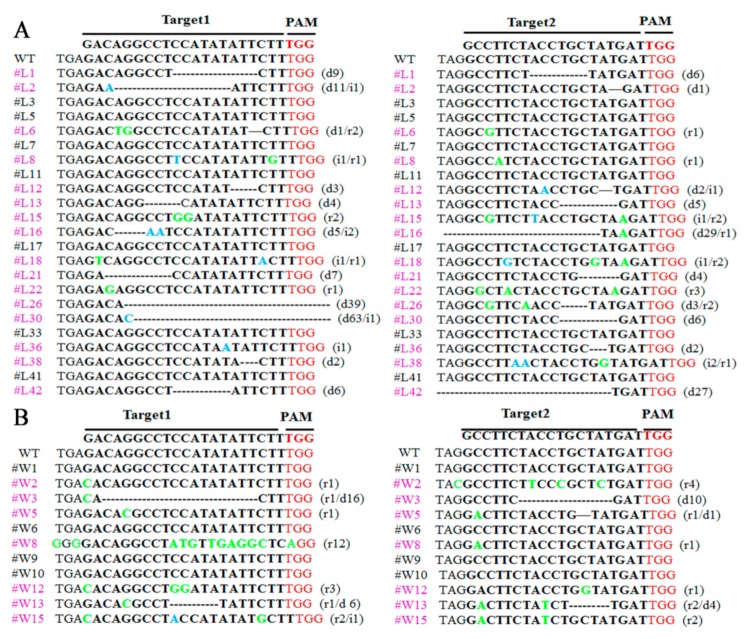
Different types of mutations detected in transgenic *Lilium* plants after CRISPR/Cas9-mediated gene editing. PAM, red. Bold, target sequence. r/green, replacements. i/blue, insertions. d/-, deletions. The purple font indicates the presence of mutant lines. Black fonts indicate lines that have not been mutated. #L, PCR-positive lines of *Lilium pumilum* DC. Fisch. #W, PCR-positive lines of ‘White Heaven’. **A** Sequencing results of *Lilium pumilum* DC. Fisch. **B** Sequencing results of ‘White Heaven’.

To analyze target site mutations in PCR-positive plants, the specific primers pdst-f/r across the target points were used to amplify a target fragment of 1453 bp for sequencing. The results showed that 16 lines of *L. pumilum* had different mutations at the target sites (Figure 7A, purple font). Among 11 transgenic lines of ‘White Heaven’, 7 lines exhibited mutations at the target site (Figure 7B, purple font), and the editing efficiency based on the obvious phenotype were 69.57% (16/23) of *L. pumilum* and 63.64% (7/11) of ‘White Heaven’.

## 3. Discussion

*Agrobacterium*-mediated genetic transformation is the most widely used transformation method. *Lilium* is difficult to transform genetically, and only a few *Lilium* transformations have been achieved thus far [8,9,10,11,12,13,47,48,49], which is largely due to a high rate of chimerism and low transformation efficiency. It has been reported that genetic transformation of *Lilium* is greatly affected by the genotype of the plant as well as the type and status of the receptor tissue used for *Agrobacterium* infection [50]. Because embryogenic callus is composed of a large number of embryogenic cells, and every single cell has the potential to develop into a somatic embryo, using embryogenic callus as a receptor material would in all probability reduce or even avoid chimerism. Moreover, the genetic stability of the transformed offspring was high, and the mutation rate was low. In this study, the embryogenic callus of *L. pumilum* was used as a receptor material for *Agrobacterium*-mediated genetic transformation for the first time. The transformation efficiency of *L. pumilum* was significantly improved reaching 29.17%. Compared to somatic embryogenesis, transformation via adventitious bud regeneration has the advantage of taking less time. In this research, the scales of ‘White Heaven’ were also used as an infection receptor material; the transformation efficiency was lower, but transformed plants could be obtained within one month. As reported previously, different receptor materials have distinct advantages and disadvantages [51]. With embryogenic callus as a recipient, high cell proliferation, high transformation efficiency, and few chimeras may be obtained, although the transformation cycle is relatively long. In contrast, the use of vegetative tissue scales as transformation recipient represents a relatively simple operation and requires much less time, although its disadvantages are lower transformation efficiency and many chimeras.

Transformation conditions are key factors affecting genetic transformation. It is generally believed that the pre-culture of explants before transformation can promote cell division, make it easier to integrate exogenous genes, and improve the level of transient expression and transformation rate of exogenous genes [52,53]. Different explants have different pre-culture durations. In this research, pre-culture of embryogenic callus for 10 days performed the best while pre-culture of scale for 3 days performed the best. Infection duration and bacterial concentration are also very important in the transformation process. Infection is a process in which *Agrobacterium* cells attach themselves to the receptor materials. *Agrobacterium* may not fully contact the receptor tissues if the infection duration is too short. However, the receptor materials might become very fragile if the infection duration is too long. Similarly, a low concentration of bacterial solution may not provide sufficient *Agrobacterium* to ensure effective infection, while a high concentration might lead to a rapid increase in bacterial growth, causing excessive damage to the receptor materials [54,55]. In this study, the transformation efficiencies with different bacterial concentrations and infection durations were significantly different. Co-culture durations and gene transfer and integration into the plant genome via *Agrobacterium* vary widely, e.g., from several hours to several days, depending on plant species, explant type and culture conditions [52,56]. It has been reported that co-culture has significant effects on the number of GUS-positive calli in rice (3 days) [52], *Lycopersicon esculentum* (3–4 days) [57], *Rosa chinensis* (2 days) [58], *Epipremnum aureum* (5 days) [59] and *Gladiolus hybridus* (12 days) [53]. In this study, co-culture for 3 days had the best effect, and transformation efficiency could not be improved by prolonging co-culture duration in *Lilium* transformation.

Due to its easy operation, low cost and high universality, CRISPR/Cas9 technology quickly became the focus of the field of genome editing once it appeared in early 2013, and it was immediately applied to basic theoretical research and the practice of crop improvement. Creation of gene mutations through genome editing or base editing by CRISPR/Cas9 has become a routine experiment. It is necessary to acquire stable non-transgenic mutants for both exact gene function research and crop improvement, especially in crop breeding, as the offspring of plants edited by CRISPR/Cas9 can be considered “non-GM” (no genetic modification) and, thus, exempt from regulatory approval [60]. For this reason, the breeding prospects of CRISPR/Cas9 technology have led many scientific research institutions and biological enterprises to work toward the establishment and optimization of genetic transformation systems. In this study, after establishing two genetic transformation systems, we successfully applied the CRISPR/Cas9 technique to *Lilium* for the first time by targeting the *PDS* reporter gene.

In conclusion, this study established two well-optimized genetic transformation systems for *Lilium*, which will lay an important foundation for functional genomic research and molecular breeding in *Lilium*. The successful application of CRISPR/Cas9 technology to *Lilium* has demonstrated the effectiveness of these two transformation systems and will have revolutionary effects on genetic trait improvement and basic theoretical research in *Lilium*.

## 4. Materials and Methods

### 4.1. Plant Materials, Agrobacterium Strain and Plasmid

*Lilium pumilum* DC. Fisch. embryogenic calli and *Lilium longiflorum* ‘White Heaven’ tissue culture seedling scales were chosen as receptor materials for genetic transformation. Embryogenic calli were obtained according to a previously described method [61]. Culture conditions were a 16/8-h light/dark cycle with a temperature of 25 ± 2 °C in the light phase (36 ± 10 μmol/m^2^/s). For media recipes, refer to Appendix Table A1. In this study, *Agrobacterium tumefaciens* EHA105 and the binary vector pCAMBIA1301 were used. The binary vector pCAMBIA1301 contains the *hygromycin (Hyg)* gene and *GUS* reporter gene. The CRISPR/Cas9 gene editing vector pBUE411 was provided by Xing [62]. Vector map reference Supplementary Figure S2. 

### 4.2. Selection Antibiotic Sensitivity Assay

Different concentrations of Hyg (0, 10, 20, 25, 30, 40, and 50 mg·L^−1^) were added to the medium for somatic embryo induction and adventitious bud differentiation, and suitable Hyg concentrations were screened for the receptor materials.

Inhibition test of cephalosporin against *Agrobacterium*: 20 μL of *Agrobacterium* tumefaciens EHA105 in overnight culture was added to YEB (Yeast Extract Mannitol Broth) liquid culture containing different concentrations of Cef (0, 100, 150, 200, 250, 300, 350, 400, 500 mg·L^−1^) After 48 h of incubation, the OD_600_ value was determined by ultraviolet (UV) spectrophotometer. The optimal concentration of Cef was determined by using YEB liquid medium without antibiotics as control. The sensitivity assay of Cef to the inoculated receptor materials was performed on the medium for somatic embryo induction and adventitious bud differentiation with different Cef concentrations (0, 100, 200, 300, 350, 400, and 500 mg·L^−1^). The antibiotic sensitivity test processed 60 explants each concentration, and each treatment was performed in three biological replicates.

### 4.3. Preparation of Agrobacterium

The plasmid pCAMBIA1301 was transformed into the strain EHA105, and positive transformants were screened by antibiotics (50 mg·L^−1^ rifampicin (Rif) and 50 mg·L^−1^ kanamycin (Kan)) and identified by PCR. A positive transformant was picked and inoculated in 5 mL fresh YEB liquid medium containing 50 mg·L^−1^ Rif and 50 mg·L^−1^ Kan at 200× *g* for 12 h. The extract was transferred to 50 mL liquid YEB medium at a ratio of 1:50 and then cultured until the OD_600_ was 0.6, and then, the culture was centrifuged at 5000× *g* for 10 min. The resuspension solution was used to resuspend the bacteria, and the resuspension was cultured at 200× *g* for 2 h as the infection solution.

### 4.4. Optimization of Agrobacterium-Mediated Transformation Conditions

To improve transformation efficiency, the key factors affecting transformation efficiency were optimized, including pre-culture duration (0, 3, 5, 7, 10, 15, and 30 days and 0, 1, 2, 3, and 4 days), *Agrobacterium* concentration (OD_600_ = 0.4, 0.5, 0.6, 0.8, and 1.0), infection duration (5, 10, 15, 20, 25 min) and co-culture duration (1, 2, 3, 5, and 7 days). The rate of *GUS* expression after screening for 10 days and the rate of resistance in the calli and buds after 2 months of resistance screening were considered. Thirty explants were used for *GUS* staining, and 60 explants were used for resistant callus buds. All data are expressed as means ± standard deviation of triplicate samples. Combining the above factors, the optimum transformation conditions were obtained.

### 4.5. Agrobacterium Transformation

The pre-cultured receptor materials were immersed in an infection solution of *Agrobacterium* for 15 min, and the tissue mass was gently shaken to ensure full contact with the infection solution. After the explants were removed from the infection medium, the residual bacteria on the surface of the tissue blocks were adequately absorbed by sterile filter paper and transferred to the co-culture medium. The co-culture medium for embryogenic callus was co-culture I, and the co-culture medium for scales was co-culture II. The continuous dark culture was carried out at 25 ± 1 °C for three days. The embryogenic calli were transferred to Selection I after co-culture, screened in dark conditions, and then transferred every two weeks. After four weeks of culture, resistant embryos were generated from the resistant calli, and any necrotic tissues at the bases of the calli were removed. The resistant embryos were transferred to germination medium. The mature somatic embryos were transferred to MS medium for further culture. After two weeks of culture, complete resistant plants were formed. After coculture, the scales were transferred to Selection III, and the screened resistant buds were transferred to MS medium for further culture under a 16/8-h light/dark cycle with a temperature of 25 ± 2 °C conditions to form complete plants.

### 4.6. Identification of Transgenic Plants

GUS histochemical assay: according to the method from Jefferson [63], the leaves, scales and roots of transformed plants were stained with GUS staining solution, and WT plants were used as controls. The staining results were observed and counted.

PCR assay: genomic DNA was extracted from successfully stained and untransformed plants using the cetyltrimethyl ammonium bromide (CTAB) method described previously, with some modifications [64]. The length of the amplified GUS fragment was 269 bp. Plasmid pCAMBIA1301 and non-transgenic plant DNA were used as positive and negative controls, respectively. The reaction system volume was 25 µL, and it included 12.5 µL LA Premix Taq (Takara, Dalian, China), 0.8 µL of 10 µM forward primer (refer to the Appendix Table A2 for primer sequences), 0.8 µL of 10 µM reverse primer, 1 µg DNA, and ddH_2_O up to 25 µL. The reaction procedure included predenaturation at 94 °C for 5 min; 30 cycles at 94 °C for 30 s, 58 °C for 42 s, and 72 °C for 30 s; and final extension at 72 °C for 2 min. The amplified products were analyzed by 1.0% agarose gel electrophoresis.

Southern blot analysis: Southern blotting was carried out using PCR-positive plants. Using plasmid pCAMBIA1301 as a template, the probe was prepared by PCR amplification with the primers GUS-F and GUS-R. The specific procedures were performed according to the instructions of the DIG (Digoxigenin) DNA Labeling and Detection Kit II (Roche diagnostics, Indianapolis, IN, USA). Five micrograms of genomic DNA was digested overnight by the restriction endonucleases EcoR I and Hind III (Takara, Dalian, China) at 37 °C. The digested DNA was separated by 0.8% agarose after enzyme digestion. The samples were transferred to Hybond N^+^ nylon membranes (Roche diagnostics, Indianapolis, IN, USA) using denaturation, neutralization and salt transfer. The membrane was hybridized with DIG-labeled specific probe at Hybridizer with 26 °C for 18 h and visualized by NBT/BCIP staining.

### 4.7. Construction of LpPDS CRISPR/Cas9 Targeting Vector and Transformation in Lilium

In our previous study, we sequenced the full-length transcripts from different tissue parts of *Lilium pumilum* DC. Fisch. plant in vitro using the Pacific Biosciences (Frasergen, Wuhan, China) single-molecule real-time (SMRT) technology. The cDNA sequences including 5′and 3′UTR of *LpPDS* were searched out from the sequence library. After predicting the ORF, two 20-nt target sequences were screened in the former central of its CDS region. Usually, the two targets strategy is likely to delete the large fragments between the two target sequences, so the mutation degree of the target gene is very high, and it is more likely to make the target gene lose its function completely. The primers were designed for synthesis, and the *Bsa*I site was introduced into the primers. Four primers, LpPDS-BsF/-BsR (10 µM) and LpPDS-F0/-R0 (0.5 µM), were mixed for the PCR assay with plasmid pMDC-T1T2 [62] as a template. The reaction system volume was 50 µL, and it included LA Premix Taq 25 µL, four primers 2 µL, 1/40 pMDC-T1T2 2 µL, ddH_2_O 21 µL. The reaction procedure included 94 °C for 5 min; 30 cycles of 94 °C for 15 s, 60 °C for 30 s, and 72 °C for 40 s; and final extension at 72 °C for 5 min. The purified PCR product LpPDS (P) and the pBUE411 vector were digested by the restriction endonuclease *Bsa*I (Gene, Hong Kong, China). The reaction system included 1 µL *Bsa*I, 1 µL 10 × CutSmart Buffer, and 8 µL pBUE411/LpPDS (P), and digestion was performed at 37 °C for 2 h. The digested pBUE411 and LpPDS (P) were added to the ligation system of 2 µL pBUE411, 3 µL LpPDS (P), and 5 µL Solution I, and the mixture was incubated at 16 °C for 2 h. The ligation mixture was transformed into *Escherichia coli* DH5α (Tiangen, Beijing, China) by the heat shock method and cultured on LB plates containing 100 mg·L^−1^ Kan overnight at 37 °C. Propagation of a single colony was performed and validated by PCR using the vector-specific primer pair OsU3-FD3 and TaU3-RD. The positive bacterial clones were sent to BGI Tech (Beijing, China) for sequencing, and the correct plasmids were transformed into *Agrobacterium tumefaciens* EHA105 and further transformed into embryogenic calli of *L. pumilum* and scales of ‘White Heaven’. The transformation method was the same as that used for the vector pCAMBIA1301, and Selection II and IV were the medium used for embryogenic calli and scale screening.

### 4.8. Identification of Transgenic Plants and Detection of Target Edit Types

Phenotype observation and molecular identification of transgenic plants were carried out. The genomic DNA of plants with an obvious phenotype was extracted for PCR using resistance marker gene primers (Bar-F and Bar-R) and primers specific for the sgRNA expression cassette (OsU3-FD3 and TaU3-RD). The target fragments were 433 bp and 831 bp, respectively. The non-transformed plants were used as controls. The target gene fragments containing the target sites were amplified using the specific primers pdst-f/r with the genomic DNA of PCR-positive plants as a template. The PCR products were sequenced, and the DNAMAN 8.0 software (Lynnon Biosoft, San Ramon, CA, USA) was used for nucleic acid sequence alignment to analyze the mutation types of the transgenic plants.

### 4.9. Statistical Analysis

Data were analyzed by analysis of variance (ANOVA) with IBM SPSS Statistics 21 software, and significant differences were observed at *p* < 0.05. All data are presented as the means ± standard error (SE) of at least three independent experiments.

*GUS* transient expression rate = *GUS*-positive receptor number/total number stained × 100%. Browning rate = number of browned explants/total explants × 100%. Proliferation rate = number of proliferated explants/total explants × 100%. Induction rate = number of inducted explants/total explants × 100%. Resistance rate = number of resistant explants/total explants × 100%.

## Figures and Tables

**Table 1:** Effects of hygromycin (Hyg) concentration on *Lilium pumilum* DC. Fisch. and ‘White Heaven’.

Variety	Concentration of Hyg (mg·L^−1^)	Rate of Browning Explants (%)	Rate of Proliferation or Induction (%)
*Lilium pumilum* DC. Fisch.	0	0.83 ± 1.44 e	99.17 ± 1.44 a
	10	16.67 ± 1.44 d	80.83 ± 3.82 b
	20	66.67 ± 2.89 c	68.33 ± 3.82 c
	30	83.33 ± 3.82 b	25.00 ± 2.50 d
	40	97.50 ± 2.50 a	4.17 ± 2.89 e
	50	100 a	0 e
‘White Heaven’	0	1.11 ± 0.58 e	92.33 ± 0.58 a
	10	24.44 ± 2.08 d	70.33 ± 2.08 b
	20	58.89 ± 3.06 c	41.11 ± 3.51 c
	30	76.67 ± 2.00 b	20.78 ± 1.53 d
	40	92.22 ± 0.58 a	7.22 ± 0.58 e
	50	100 a	0 e

Note: Statistically, one-way analysis of variance (ANOVA) and Duncan’s post-hoc tests were used for the comparison each treatment. Different letters indicate significant difference within a group as determined by Student’s *t*-test (*p* < 0.05). The same is true for Table 2.

**Table 2:** Influence of cefotaxime (Cef) on the proliferation of *Lilium pumilum* DC. Fisch. and ‘White Heaven’.

Variety	Concentration of Cef (mg·L^−1^)	Rate of Browning Explants (%)	Rate of Proliferation or Induction (%)
*Lilium pumilum* DC. Fisch.	0	0.83 ± 1.44 g	100 a
	100	6.67 ± 1.44 f	96.67 ± 3.82 ab
	200	12.50 ± 2.50 e	91.67 ± 3.82 b
	300	20.00 ± 2.50 d	85.00 ± 2.50 c
	350	32.50 ± 2.50 c	80.00 ± 2.50 c
	400	50.00 ± 2.50 b	71.67 ± 3.82 d
	500	74.17 ± 2.89 a	61.67 ± 3.82 e
‘White Heaven’	0	0 f	100 a
	100	5.22 ± 0.58 e	91.11 ± 1.53 b
	200	17.56 ± 1.15 d	83.33 ± 1.00 c
	300	24.89 ± 1.00 c	75.56 ± 0.58 d
	350	32.33 ± 1.53 b	70.00 ± 1.73 d
	400	39.56 ± 1.15 b	57.78 ± 1.53 e
	500	47.89 ± 1.53 a	52.22 ± 0.58 e

## Supplementary Materials

Supplementary materials can be found at https://www.mdpi.com/1422-0067/20/12/2920/s1.

## Abbreviations

MS (Culture medium)	Murashige-Skoog
PIC (IAA analogue)	Picloram (Sigma-Aldrich, United States)
NAA (Auxin analogue)	Naphthylacetic acid (Sigma-Aldrich, United States)
6-BA (Cytokinin)	N6-benzyladenine (Tiangen, Beijing, China)
AS (Chemical material)	Acetosyringone (Tiangen, Beijing, China)
Cef (Antibiotic)	Cefotaxime (Tiangen, Beijing, China)
Hyg (Antibiotic)	Hygromycin phosphotransferase (Sigma-Aldrich, United States)
Kan (Antibiotic)	Kanamycin monosulfate (Tiangen, Beijing, China)
Rif (Antibiotic)	Rifampicin (Tiangen, Beijing, China)
Bar	Basta resistance gene
GUS	β-glucuronidase

## Appendix A

**Table A1:** Media used for *Lilium* explant transformation.

Media	Composition
*Lilium pumilum* DC. Fisch.	
Pre-cultivation I	MS (Murashige-Skoog) + 1.0 mg·L^−1^ PIC (Picloram) + 0.2 mg·L^−1^ NAA (Naphthylacetic acid) + 30 g·L^−1^ Sucrose + 7.0 g·L^−1^ Agarose
Co-cultivation I	MS (NH_4_NO_3_-free) + 1.0 mg·L^−1^ PIC + 0.2 mg·L^−1^ NAA +100 μmol AS (Acetosyringone) + 60 g·L^−1^ Sucrose + 7.0 g·L^−1^ Agarose
Selection I	MS + 1.0 mg·L^−1^ PIC + 0.2 mg·L^−1^ NAA + 400 mg·L^−1^ Cef (Cefotaxime) + 30 mg·L^−1^ Hyg (Hygromycin) + 30 g·L^−1^ Sucrose + 7.0 g·L^−1^ Agarose
Selection II	MS + 1.0 mg·L^−1^ PIC + 0.2 mg·L^−1^ NAA + 400 mg·L^−1^ Cef + 10 mg·L^−1^ Basta +30 g·L^−1^ Sucrose + 7.0 g·L^−1^ Agarose
Somatic embryo germination medium	MS + 0.5 mg·L^−1^ 6-BA (N6-benzyladenine) + 30 g·L^−1^ Sucrose + 7.0 g·L^−1^ Agarose
‘White Heaven’	
Pre-cultivation II	MS + 1.5 mg·L^−1^ BA + 0.2 mg·L^−1^ NAA + 30 g·L^−1^ Sucrose + 7.0 g·L^−1^ Agarose
Co-cultivation II	MS (NH_4_NO_3_-free) + 1.5 mg·L^−1^ BA + 0.2 mg·L^−1^ NAA +100 μmol AS + 60 g·L^−1^ Sucrose + 7.0 g·L^−1^ Agarose
Selection III	MS (NH_4_NO_3_-free) + 1.5 mg·L^−1^ BA + 0.2 mg·L^−1^ NAA + 400 mg·L^−1^ Cef + 40 mg·L^−1^ Hyg +30 g·L^−1^ Sucrose + 7.0 g·L^−1^ Agarose
Selection IV	MS (NH_4_NO_3_-free) + 1.5 mg·L^−1^ BA + 0.2 mg·L^−1^ NAA + 400 mg·L^−1^ Cef + 20 mg·L^−1^ Basta + 30 g·L^−1^ Sucrose + 7.0 g·L^−1^ Agarose
Resuspension solution	½MS (NH_4_NO_3_-free) + 100 μmol AS + 60 g·L^−1^ Sucrose

**Table A2:** Primers used in the study.

Primer Name	Primer Sequence (5′-3′)
GUS-F	AGTTCTTTCGGCTTGTTG
GUS-R	TTCTACTTTACTGGCTTTGG
Hyg-F	TACACAGCCATCGGTCCAGA
Hyg-R	CGCAAGGAATCGGTCAATACAC
Bar-F	CTGCACCATCGTCAACCACTAC
Bar-R	CTGCCAGAAACCCACGTCAT
LpPDS-F0	GACAGGCCTCCATATATTCTTGTTTTAGAGCTAGAAATAGC
LpPDS-R0	ATCATAGCAGGTAGAAGGCCGCTTCTTGGTGCC
LpPDS-BsF	AATAATGGTCTCAGGCGACAGGCCTCCATATATTCTT
LpPDS-BsR	ATTATTGGTCTCTAAACATCATAGCAGGTAGAAGGC
OsU3-FD3	GACAGGCGTCTTCTACTGGTGCTAC
TaU3-RD	CTCACAAATTATCAGCACGCTAGTC
pdst-f	CTGAGTTTCGGAGTCGTG
pdst-r	CCAGCCAGATAGAACCCT
