# Supplementary material for: Establishment of Efficient Genetic Transformation Systems and Application of CRISPR/Cas9 Genome Editing Technology in Lilium pumilum DC. Fisch. and Lilium longiflorum White Heaven
Source: Int J Mol Sci. 2019 Jun 14;20(12):2920. doi: 10.3390/ijms20122920 (PMC6627044; doi:10.3390/ijms20122920)
Supplement: Supplementary file 1 [file ijms-20-02920-s001.pdf]

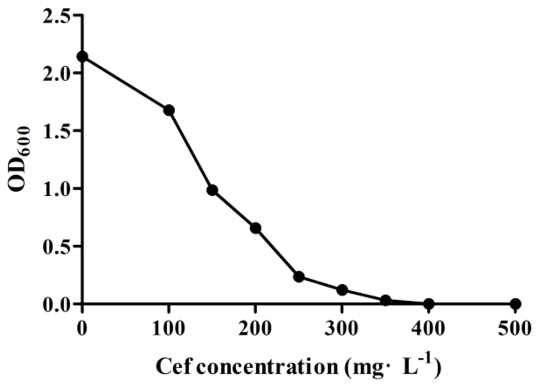

Figure S1. Influence of Cef on *Agrobacterium* EHA105

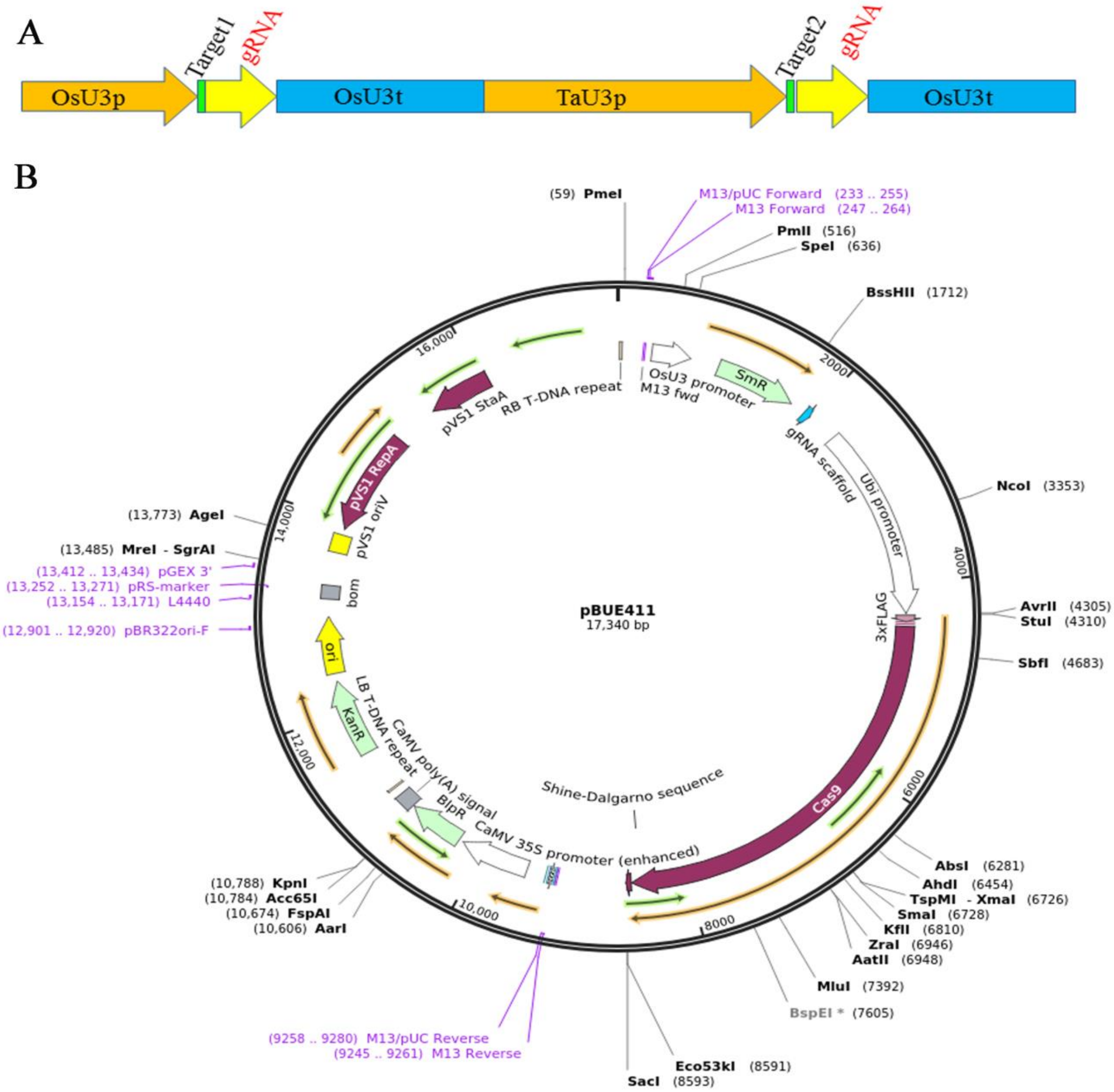

C Sequence for *LPPDS*

GTCCTTCCTCGGTGGAATCAAGCGTTCCCGCACTCGCCGCACCCTATCCCGCCCGTCTGTCGCTACCTCCACC
AGAATCCTTTCCACTGATCCCCTCACCAATTTCTCTCTCTAAAACTCCAAAACCCCACTAAGGGACTCACAAA
TTCGATCCTCAACAATCGGTTCTCTTCGTCATGAACTCCGCCGGATCGATCTCCACCGTTTGCCATCACCGGC
AGGTATCCAAGATCTTCCATCAGTAGCTACTGATAGCATCAATTTCAGCTCGAGAGCGCTTCAATTCATGGCG
CGGAGACGACATTGACGCGGAGAGTCGCCGGTCCCTTGCAGGTTGTTTGCAAGGATTGTCCGAGGCCGCGAGC

TCGATATACGGTGAATTTTCTTGAAGCGGCTAAGTTGTCGGCCTGAGTTTCGGAGTCGTGCAAGACAGGAGAA
GCCGCTGCCAAGTTGTGATTGCCGGGGCCAGGTCTGGCGGGTTTATCTACGGCCAAAGTATCTTGCCGATGCA
GGACATATACCTATACTGTTGGAGGGGAGAGATGTTTGGGTGGAAAGATTGCTGCATGGAAGGATAGTGATGG
AGATTGGTATGAG**ACAGGCCTCCATATATTCTT**TGGAGCTTATCCCAATGTGCAGAATTTGTTTGGGG
AACTTGGTATTAATGATCGTTTGCAGTGGAAGGAGCACTCTATGATTTTGCAATGCCAAACAAGCCAGGAGAA
TTTAGCCGCTTTGATTTTCCAGAGGTTCTTCCTGCACCCTTAAATGGAATATGGGCCATCTTAAAGAATAATG
AAATGCTGACCTGGCCAGAAAAAGTGCGTTTTGCCATA**GGCCTTCTACCTGCTATGAT**TGGGGGACAG
GCCTATGTTGAGGCTCAGGATGGTTTAACTGTTAAAGAGTGGATGAGGAAGCAGGGCGTTCCTGAACGTGTCA
ATGACGAAGTTTTCATTGCAATGTCCAAAGCTCTTAATTTTATAAATCCAGATGAGCTTTCCATGCAGTGCAT
TTTAATTGCTTTAAACCGTTTTCTTCAGGAAAAGCATGGCTCCAAGATGGCTTTCTTAGATGGTAATCCCCCC
GAGAGGTTGTGCATACCAATTGTTGACCACATTCAATCATTGGGTGGTCAAGTCCTACTTAACTCTCGTATAC
AGAAAATTGAGTTGAATTCTGATAGTACTGTGAAAAACCTTATACTGAGCAATGGGAAAATAATCAATGGAGA
TGTTTATGTAATAGCTACTCCTGTTGATGTCTTGAAGCTTCTTTTGCCTCAAGAATGGAGAGAAATTTCATAC
TTCAAGAAATTGGATAAATTGGTGGGAGTTCCTGTGATCAATGTTCATATATGGTTTGACAGGAAACTGAAGA
ACACCTATGATCATCTTCTTTTCAGCAGGAGTCCTCTTTTGAGTGTTTATGCAGATATGTCTGTAACTTGCAA
GGAATATTACGATCCTTATCGTTCTATGCTTGAGTTGGTATTCGCACCGGCAGAGGAATGGATCTCACGTAGT
GACGATGAGATCATCGATGCTACAATGACAGAACTGCTAAGCTGTTCCCAGATGAAATAGCCGCAGATCAGAG
TAAAGCGAAAATTCTCAAGTATCATGTTGTCAAAACTCCAAGATCTGTTTACAAAACTGTGCCCGACTGTGAA
CCGTGCCGGCCTCTGCAAGATCGCCAATTGAAGGGTTCTATCTGGCTGGCGATTACACGAAGCAGAAGTACTT
GGCTTCGATGGAGGGGGCTGTTTTGTCTGGGAAGCTTTGTGCGCAGTCTATTGTACAGGATTACGACATTTTA
GTTGATCGGACGAAGAGAAGCCCTCAAGCCGAGATGTCAGTCGTC**TAG**TTGCGGAATTTGGAGTCATAGCAGA
CATCTTTGCTATACAATGTATTCTTTGCGTGCTGAGAAAATCAGATCTCAGAATATATACATTAAAAAATGGT
AGATTTTCTCTGAGTGGGTTAGAGCATGTCACCCCATTGGATTATATCGTAGATATGAAATATTTGAAGCAAA
TTACAATTACATACATGTCGAT

Figure S2. *LpPDS* CRISPR / Cas9 targeted vectors. **A** is a schematic representation of the integration of two targets into two gRNA expression cassettes. **B** is a map of the gene editing vector pBUE411. **C** is the reference sequence for *LpPDS*, with the target highlighted in yellow and red font showing the PAM sequence.
